# Role of Motif III in Catalysis by Acetyl-CoA Synthetase

**DOI:** 10.1155/2012/509579

**Published:** 2012-08-15

**Authors:** Cheryl Ingram-Smith, Jerry L. Thurman, Karen Zimowski, Kerry S. Smith

**Affiliations:** Department of Genetics and Biochemistry, Clemson University, Clemson, SC 29634-0318, USA

## Abstract

The acyl-adenylate-forming enzyme superfamily, consisting of acyl- and aryl-CoA synthetases, the adenylation domain of the nonribosomal peptide synthetases, and luciferase, has three signature motifs (I–III) and ten conserved core motifs (A1–A10), some of which overlap the signature motifs. The consensus sequence for signature motif III (core motif A7) in acetyl-CoA synthetase is Y-X-S/T/A-G-D, with an invariant fifth position, highly conserved first and fourth positions, and variable second and third positions. Kinetic studies of enzyme variants revealed that an alteration at any position resulted in a strong decrease in the catalytic rate, although the most deleterious effects were observed when the first or fifth positions were changed. Structural modeling suggests that the highly conserved Tyr in the first position plays a key role in active site architecture through interaction with a highly conserved active-site Gln, and the invariant Asp in the fifth position plays a critical role in ATP binding and catalysis through interaction with the 2′- and 3′-OH groups of the ribose moiety. Interactions between these Asp and ATP are observed in all structures available for members of the superfamily, consistent with a critical role in substrate binding and catalysis for this invariant residue.

## 1. Introduction

AMP-forming acetyl-CoA synthetase (Acs, EC 6.2.1.1), which catalyzes the formation of acetyl-CoA from acetate, ATP, and CoASH (acetate + ATP + CoASH ⇆ acetyl-CoA + AMP + PP_i_), belongs to the acyl-adenylate-forming enzyme superfamily, which has newly been designated by Gulick [1] as the ANL superfamily of adenylating enzymes to reflect the three subfamilies, the acyl- and aryl-CoA synthetases, the adenylation domain of the nonribosomal peptide synthetases, and luciferase. Although distant members of this superfamily catalyze wholly unrelated reactions and employ different substrates, they share the property of formation of an enzyme-bound acyl-adenylate intermediate in the first step via activation with ATP with concurrent release of pyrophosphate. 

Sequence alignment of members of this superfamily has revealed the presence of three signature motifs as defined by Chang et al. [2]:

  motif I: **T**[S/G]-**S**[G]-[G]-[S/T]-**T**[S/E]-**G**[S]-[X]-**P**[M]-[K]-**G**[L/F],
  motif II: **Y**[L/W/F]-**G**[S/M/W]-X-**T**[A]-**E**,
  motif III: **Y**[F/L]-**R**[T/K/X]-**T**[S/V/A]-**G**-**D**,

(boldfaced residues are the predominant residue at each position, and alternative residues are indicated in bracket.)

Marahiel et al. [3] further identified ten conserved core motifs in the superfamily, in which the A3, A5, and A7 motifs overlap with or encompass motifs I, II, and III, respectively. All three of these motifs are located in or near the active site of each enzyme. Motifs I and II have been shown to play roles in formation of the adenylate based on evidence from enzymes altered at positions within these motifs [2, 4–13]. However, motif III is less well conserved and has received much less attention. 

Here, we have investigated the role of motif III in acetyl-CoA synthetase (Acs) in the *Methanothermobacter thermautotrophicus* Acs1 (Acs1_Mt_). This recombinant enzyme has been previously characterized and shows a strong preference for acetate as the acyl substrate and ATP as the nucleotide triphosphate [4, 14], typical of most Acs enzymes. Our results indicate an important role for motif III in catalysis as alteration of any position resulted in a strong decrease in the turnover rate. The highly conserved Tyr in the first position may play a key role in active-site architecture through interaction with a highly conserved active-site Gln. The invariant Asp in the fifth position plays a critical role in ATP binding and catalysis through interaction with the 2′- and 3′-OH groups of the ribose moiety of ATP. The role of this residue in Acs is discussed further in the context of its role in other members of the superfamily.

## 2. Experimental Procedures

### 2.1. Materials

Chemicals were purchased from VWR Scientific Products, Fisher Scientific, or Sigma Chemicals. Oligonucleotides for site-directed mutagenesis were purchased from Integrated DNA Technologies. IRD-700- and IRD-800-labeled oligonucleotides for DNA sequencing were purchased from Li-Cor Biosciences or MWG Biotech. 

### 2.2. Sequence Alignment

 Sequence alignments were performed using Clustal X [15] with a Gonnet PAM 250 weight matrix and the default parameters of 10.0 and 0.05 as the gap opening and gap extension penalties, respectively. 

### 2.3. Site-Directed Mutagenesis

 Site-directed mutagenesis of the gene-encoding *M. thermautotrophicus* Acs1 was performed using the QuikChange Site-Directed Mutagenesis Kit (Stratagene). Mutagenic primers were approximately 40 nucleotides, with the altered site located at the center. Mutations were confirmed by bidirectional DNA sequencing using the Thermo Sequenase Primer Cycle Sequencing Kit (GE Healthcare) at the Nucleic Acid Facility at Clemson University. 

### 2.4. Heterologous Production and Purification of Acs1_Mt_ Variants

Unaltered Acs1_Mt_ and its variants were heterologously produced in *Escherichia coli* Rosetta Blue (DE3) (Novagen) as described previously [4, 14]. Cells harboring the Acs1_Mt_ expression construct were grown at 37°C to an A_600_ of ~0.6, and enzyme production was induced by the addition of IPTG to a final concentration of 0.5 mM. Cell growth was continued overnight at ambient temperature, and cells were then harvested. Acs1_Mt_ and variant enzymes were purified by a two-step procedure employing Q-sepharose anion exchange and phenyl sepharose hydrophobic interaction chromatography as previously described [4, 14]. An additional Source Q anion exchange chromatography step was added if the variant enzyme was not sufficiently pure after the first two steps. The purified enzymes were dialyzed and concentrated, and aliquots were stored at −20°C. Protein concentration was determined by the Bradford method [16].

### 2.5. Molecular Mass Determination of Enzyme Variants

 The variants were subjected to gel filtration chromatography to determine subunit composition. A Superose 12 gel filtration column (GE Healthcare), preequilibrated with 50 mM Tris [pH 7.5] containing 150 mM KCl, was calibrated with chymotrypsinogen (25 kDa), ovalbumin (43 kDa), albumin (67 kDa), aldolase (158 kDa), catalase (232 kDa), ferritin (440 kDa), and blue dextran (2000 kDa). 

### 2.6. Enzymatic Assay for ACS Activity

Enzymatic activity was determined by the hydroxamate assay, which monitors formation of activated acyl groups such as the acetyl-CoA product of the ACS reaction [17, 18]. The standard reaction contained 100 mM Tris [pH 7.5], 600 mM hydroxylamine-HCl [pH 7.0], and 2 mM glutathione (reduced form) in addition to the three substrates (HSCoA, MgATP, and acetate) in a 300 *μ*L reaction volume. In all cases, the concentration of Mg^2+^ was the same as that for ATP. Reactions were performed at the optimal temperature for Acs1_Mt_ of 65°C [4, 14], terminated by the addition of two volumes of stop solution (1 N HCl, 5% trichloroacetic acid, 1.25% FeCl_3_), and the color change was measured by the change in absorbance at 540 nm.

 For determination of apparent kinetic parameters, one substrate was varied, and the other two substrates were held at a saturating concentration, generally ten times the *K*_*m*_ value. The concentration of the variable substrate ranged from ~0.2 to 5–10 times the *K*_*m*_ value. The apparent kinetic parameters *k*_cat_ and *k*_cat_/*K*_*m*_ were determined using nonlinear regression to fit the experimental data to the Michaelis-Menten equation. Each kinetic determination represents three replicates, and the standard errors are given. The enzyme variants followed Michaelis-Menten kinetics for all substrates.

### 2.7. Inhibition Assays

 Inhibition of wild-type Acs1_Mt_ by adenosine and its derivatives and ribose was determined using the hydroxamate assay. In these assays, all three substrates were held at saturating levels, and ribose or adenosine was added to the reaction mix to a final concentration ranging from 0 to 1000 mM or 0 to 100 mM, respectively. The *K*_*i*_ value for each inhibitor was determined by reciprocal plot of velocity versus inhibitor concentration.

### 2.8. Modeling Motif III Residues of Acs1_Mt_

 The structures of Acs1_Mt_ and the variants were modeled on the *S. enterica* Acs (PDB ID: 2P2F) [19] and the *S. cerevisiae* Acs1 structures (PDB ID: 1RY2) [20] by DS Modeler (Accelrys) using the default parameters. Structures were compared to the *S. enterica* and *S. cerevisiae* Acs structures to ensure the modeling did not introduce major structural alterations. 

## 3. Results and Discussion

Sequence alignment indicates considerable conservation of motif III among the Acs sequences, with the first, fourth, and fifth positions highly or completely conserved, but the second and third positions showing a higher level of variability and an overall consensus of Y-X-S/T/A-G-D (Table 1). An alignment of Acs with other members of the adenylate-forming superfamily confirms that these positions are highly conserved throughout (Table 1). The *Saccharomyces cerevisiae* ACS1 structure (Acs_Sc_; PDB 1RY2) contains AMP [20] and is in a conformation thought to catalyze the first step of the Acs reaction in which acetate and ATP are bound and an enzyme-bound acetyl adenylate is formed with concomitant release of inorganic pyrophosphate. The *Salmonella enterica* Acs structure (Acs_Se_; PDB 2P2F) [19] contains acetate, AMP, and CoA [8] and is thought to be in the conformation for catalysis of the second step of the reaction, in which the C-terminal domain is repositioned near the active site to bring new residues into context for CoA binding and formation of the acetyl-CoA product with release of AMP [8, 19, 20].

**Figure 1:**
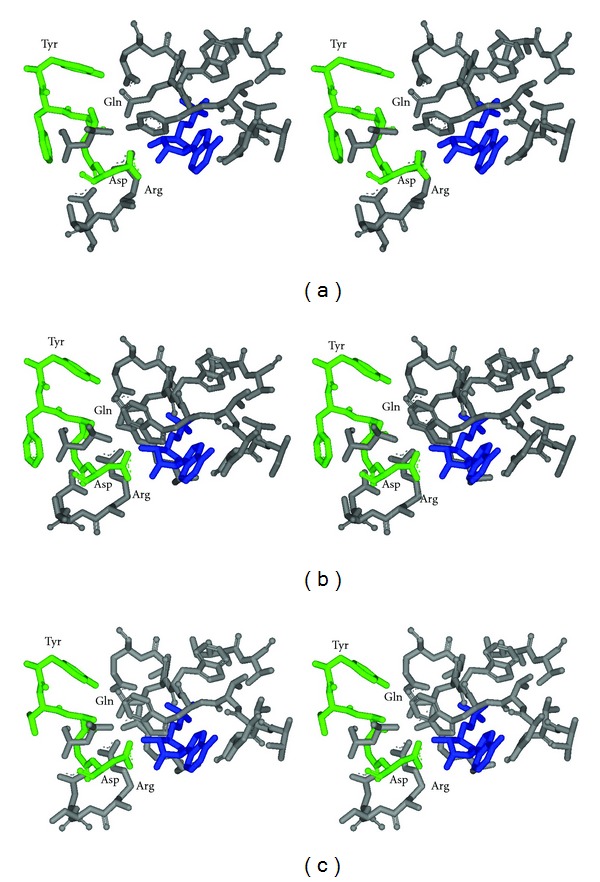
Position of motif III residues in the active sites of (a) Acs_Sc_, (b) Acs_Se_, and (c) the Acs1_Mt_ structural model. Acs1_Mt_ modeled on the Acs_Se_ structure (PDB:2P2F) using Accelrys DS Modeler and the stereo images were created using Accelrys DS ViewerPro 5.0. For clarity in viewing, only some residues within the 10Å sphere of AMP are shown. AMP is shown in blue, and the motif III residues are shown in green. Residues discussed in the text are labeled.

 Inspection of the Acs_Se_ and Acs_Sc_ structures places motif III in the active site, regardless of which conformation of the enzyme. The positioning of motif III residues near the adenylate moiety of the bound AMP ligand suggests that residues in this motif may play a role in ATP binding and/or catalysis. Modeling of Acs1_Mt_ on the *S. enterica* and *S. cerevisiae* Acs structures places the motif III residues in a similar position to interact with substrates (Figure 1).

In Acs1_Mt_, motif III has the sequence ^498^YTAGD^502^. We individually altered each position of motif III in Acs1_Mt_ and determined the kinetic parameters of the purified enzyme variants. The residues in the highly conserved first, fourth, and fifth positions were changed to either Ala or a conservative amino acid replacement. The Thr residue at the more variable second position was changed to Ala, and the Ala residue in the third position was changed to Thr, as this is the residue found in many Acs sequences. The kinetic parameters for the purified enzyme variants were determined using the hydroxamate assay and are shown in Table 2. 

The hydroxamate assay measures activated acyl groups including both acetyl-AMP and acetyl-CoA by their conversion to the acyl hydroxamate and subsequently to a ferric hydroxamate complex. Wilson and Aldrich [21] have shown that several stand-alone adenylating enzymes belonging to the same superfamily as Acs slowly release the acyl-adenylate intermediate in the absence of the native acceptor, and this released acyl-adenylate can react with hydroxylamine. Meng et al. [22] also witnessed this phenomenon with a medium chain acyl-CoA synthetase (Macs) that favors 2-methylbutyrate as the acyl substrate. In this case, Macs released the acyl adenylate to varying degrees in the absence of the CoA acceptor when a less favored acyl substrate such as propionate was used. However, little to no release of the acyl-adenylate intermediate was observed in the absence of CoA with the favored 2-methylbutryate, suggesting that the acyl-adenylate intermediate is retained if the acyl moiety fits well in the active site but is more readily released in the absence of the native acceptor if the fit is suboptimal. 

No activity was detected with Acs1_Mt_ with acetate in the absence of HSCoA, indicating that the acetyl-AMP intermediate remains enzyme bound and that the bound intermediate is not reactive with hydroxylamine. Thus, the kinetic parameters shown in Table 2 are for the overall reaction, although the *K*_*m*_ values for ATP and acetate would likely be similar if just the first adenylation step of the reaction was measured.

Several of the variants were found to be inactive over a wide range of concentrations for each substrate and a range of enzyme concentrations. Enzymes that were inactive displayed similar behavior in both the ion exchange and hydrophobic interaction chromatography steps during purification, and gel filtration chromatography indicated the variants are dimeric as for the wild type enzyme, suggesting there are no gross structural alterations. Overall, alteration of any of the residues in motif III appeared to have a strong deleterious effect on catalysis, although substrate affinity was generally not impaired.

### 3.1. Positioning of Tyr^498^ Plays an Important Role in Active-Site Architecture

Based on the two Acs structures, the highly conserved Tyr^498^ in the first position of motif III is part of a hydrogen bond network with Gln^417^ and through this hydrogen bond network may contribute to maintenance of the active-site architecture near the ATP binding site (Figures 1(a) and 1(b)). In the Acs1_Mt_ model (Figure 1(c)), there is an additional interaction between Ala^500^ and Gln^417^. To examine whether it is the hydrophobic and bulky nature of Tyr^498^ or its participation in this hydrogen bond network that plays the more important role in substrate binding and catalysis, this residue was altered to both Ala and Phe. The Tyr^498^Ala alteration in Acs1_Mt_ did not significantly affect the *K*_*m*_ for any substrate but reduced the turnover rate *k*_cat_ 41-fold (Table 2). However, the Tyr^498^ Phe variant was soluble but inactive at all substrate and enzyme concentrations tested. These results suggest that although the size of Tyr^498^ is important in maintaining active-site architecture, the hydroxyl moiety plays a critical role in properly positioning this large side chain through hydrogen bonding with Gln^417^. Attempts at chemical rescue of the Tyr^498^ Ala variant with phenol were unsuccessful.

### 3.2. Thr^499^ and Ala^500^ Are Less Well Conserved and Play Lesser Roles

 The second and third positions of motif III, represented by Thr^499^ and Ala^500^ in Acs1_Mt_, are less well conserved than the other positions (Table 1). Thr^499^ is replaced by Phe in Acs_Se_, Acs_Sc_, and many other Acs sequences, but Leu is observed in that position in four of the five Acs sequences in *Methanosaeta concilii* as well as all four of the Acs sequences in *Methanosaeta thermophila*. Ser and Thr are commonly observed at the third position in motif III, although Ala is present at this position in eight of the nine total Acs sequences in *M. concilii* and *M. thermophila*.

These positions were individually altered to Ala and Thr, respectively, in Acs1_Mt_, and the purified variants were analyzed. The *K*_*m*_ values for substrates showed only minor changes (less than threefold increase or decrease) versus the unaltered enzyme. However, the *k*_cat_ value decreased 83-fold for the Thr^499^Ala variant and 44-fold for the Ala^500^ Thr variant (Table 2). Overall, these results suggest a less important role for these positions, which is consistent with the lower level of conservation observed. 

### 3.3. Gly^501^ Is Highly Conserved and May Properly Position the Invariant Asp^502^

 Gly^501^ in the fourth position of motif III is almost completely conserved within the acyl-adenylate-forming enzyme superfamily except for a few members most distantly related to Acs. Replacement of this residue by Ala resulted in two- to threefold reduced *K*_*m*_ values for all three substrates; however, the turnover rate was over 200-fold reduced (Table 2). The strict conservation of this Gly and the reduced catalysis observed for the Gly^501^Ala variant are consistent with this residue playing a role in proper positioning of the critical Asp^502^ residue in the adjacent position.

### 3.4. Asp^502^ Plays a Critical Role in ATP Binding through Interaction with the 2′-OH of the Ribose Moiety

To investigate the role of the invariant Asp residue of motif III, Asp^502^ of Acs1_Mt_ was altered to Ala and the more conservative residues Glu and Asn. Although the enzyme variants were soluble, each of these alterations eliminated all detectable enzymatic activity, regardless of substrate concentrations or concentration of enzyme used. The fact that even the most conservative changes inactivated the enzyme indicates that this Asp is absolutely critical for activity, as might be expected since Asp^502^ is completely conserved among all ACSs and throughout the superfamily.

**Figure 2:**
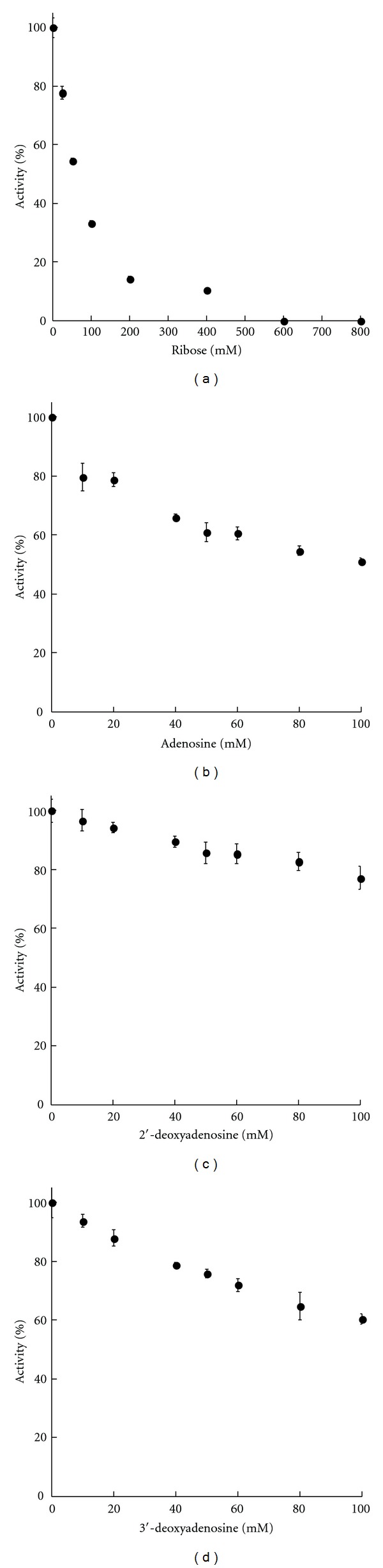
Inhibition of Acs1_Mt_. (a) Ribose, (b) adenosine, (c) 2′-deoxyadenosine, and (d) 3′-deoxyadenosine. Assays were performed with the indicated concentrations of inhibitor in the reaction, and results are plotted as a percentage of the activity observed in the absence of inhibitor, with error bars as shown. The *K*_*i*_ value for each inhibitor was determined by extrapolation of the data.

 Inhibition assays were performed as an indirect approach to delineate the interaction between Asp^502^ and ATP. Ribose completely inhibited enzyme activity at concentrations above 600 mM, and the *K*_*i*_ was determined to be 53 mM (Figure 2(a)). The maximum adenosine concentration that could be reached in inhibition assays was 100 mM, which produced partial inactivation. However, extrapolation of the data indicated a *K*_*i*_ of ~121 mM for adenosine (Figure 2(b)). That the *K*_*i*_ for ribose was approximately half the estimated *K*_*i*_ for adenosine suggests that interaction between the enzyme and the ribose moiety plays an important role in ATP binding.

 To determine more precisely the interaction between Asp^502^ and the 2′- and 3′-OH groups of the ribose sugar of adenosine, inhibition by 2′- and 3′-deoxyadenosine was examined. Although only partial inhibition was observed with either compound, extrapolation of the results gave apparent *K*_*i*_ values of 356 mM and 151 mM for 2′- and 3′-deoxyadenosine, respectively (Figures 2(c) and 2(d)). These results suggest that interaction between Asp^502^ and adenosine is mediated primarily through the 2′-OH group of the ribose sugar, as the absence of the 3′-OH group had minimal effect. 

The Acs1_Mt_ model (Figure 1(c)) predicts hydrogen bonds between Asp^502^ and both the 2′ and 3′-OH groups of the ribose moiety. However, these hydrogen bonds are eliminated in the Asp^502^Ala variant. The inhibition results and the complete impairment of enzymatic activity by alterations at Asp^502^ suggest that the interaction of Asp^502^ of motif III with the 2′-OH plays a key role although interaction with the 3′-OH is also important for achieving optimal activity.

### 3.5. Interaction between the Invariant Asp and the Ribose Moiety of ATP in Other Members of the Enzyme Superfamily

 The active-site architecture in Acs_Sc_ and Acs_Se_ is similar in the vicinity of the AMP ligand (Figures 1(a) and 1(b)). However, Asp^559^ of motif III in Acs_Sc_ interacts with both the 2′- and 3′-OH groups and with Arg^574^, whereas Asp^500^ of motif III in Acs_Se_ hydrogen bonds with the 2′-OH group and Trp^413^ and the 3′-OH interacts with Gln^415^ and Arg^515^ [8, 20]. The Acs_Sc_ structure is proposed to be that for the enzyme poised to catalyze the first step of the reaction, whereas the Acs_Se_ structure has the C-terminal domain shifted inward toward the active-site to bring additional amino acid residues into context for substrate binding and catalysis of the second step of the reaction [8, 19, 20]. Whether these differences are due to slight changes in active-site architecture between two different enzymes or a movement of this Asp residue as the enzyme converts from one conformation to the other during catalysis of the two steps is unknown, as structures in both conformations are not available for either enzyme. 

Structures have been determined for a number of enzymes spanning the adenylate-forming enzyme superfamily, including short, medium, and long-chain acyl-CoA synthetases, the aryl-CoA synthetases CBL and benzoyl-CoA ligase, several NRPS adenylation domains, and luciferase. These structures have revealed that domain alternation between the first and second steps of the reaction is universal among the superfamily [1]. Inspection of those structures with bound ligands indicates that in each case the invariant Asp in motif III/A7 interacts with one or both hydroxyl groups of the ribose moiety of ATP [6–8, 10, 19, 20, 23–29]. 

Three other members of the superfamily have structures in both the adenylate-forming and thioester-forming conformations. In 4-chlorobenzoate:CoA ligase (CBL), Asp^385^ hydrogen bonds with just the 2′-OH, whereas the 3′-OH interacts with Arg^400^ in the adenylate-forming conformation, whereas in the thioester-forming conformation, Asp^385^ maintains its hydrogen bond with the 2′-OH and now also interacts with 3′-OH group along with Arg^400^ [6, 7]. In DltA, the D-alanine:D-alanyl carrier protein ligase, Asp^383^, interacts with both hydroxyl groups in both conformations. The 2′-OH also interacts with Tyr^394^ in the adenylate-forming conformation, and the 3′-OH also interacts with Arg^396^ in the thioester-forming conformation [25, 27]. Most recently, structures for the human medium-chain acyl-CoA synthetase ACSM2A in both conformations have been reported [28]. Asp^446^ interacts with both the 2′-OH and 3′-OH in both the adenylate-forming and thioester-forming conformations for this enzyme. Thus, different enzymes interact with the ribose moiety of ATP in different ways. In some cases, the interaction changes slightly after domain alternation. However, in all cases, the invariant Asp of motif III interacts with at least the 2′-OH, suggesting that this is the most important interaction.

### 3.6. Role of the Invariant Asp in Other Members of the Enzyme Superfamily

The role of this invariant Asp has been studied biochemically in only a few members of the superfamily. In 3-chlorobenzoate-CoA ligase, alteration of this Asp to Val essentially eliminated all catalytic activity [30]. Gocht and Marahiel [31] reported that for gramicidin synthetase 1, replacement of this Asp residue with Asn or Ser reduced activity by 22% and 88%, respectively. Pavela-Vrancic et al. [32] observed with this same enzyme that replacement of ATP in the reaction with 2′-dATP resulted in a 20% reduction in activity versus a 74% reduction in activity when ATP was replaced with 3′-dATP. These results suggest that for gramicidin synthetase 1, as for Acs, interaction between the invariant Asp and the hydroxyl groups of the ribose moiety of ATP is important, with the interaction with the 2′-OH playing the most important role. 

In CBL, the invariant Asp^385^ of motif III hydrogen bonds with the 2′-OH group. Alteration of this residue to Ala greatly reduced the overall rate of catalysis, primarily due to a reduced rate for the first step of the reaction, and resulted in increased *K*_*m*_ values for both ATP and 4-chlorobenzoate [7]. In D-alanyl carrier protein ligase, the invariant Asp hydrogen bonds with both the 2′- and 3′-OH groups of the ribose moiety [27]. Alteration of this residue to Asn reduced the rate of catalysis of the adenylation reaction only twofold but resulted in a 75-fold increased *K*_*m*_ value for ATP, leading the authors to conclude that this Asp plays a major role in tight binding of ATP and the adenylate intermediate [25].

## 4. Conclusions

 The results from this investigation indicate that the motif III/A7 signature motif in Acs plays an important role in both active-site architecture and ATP binding and catalysis. 

Although all positions of this motif appear to play an important role in catalysis, the Tyr at the first position that is highly conserved among Acs sequences helps maintain active site architecture through a hydrogen bond network with other active-site residues, particularly the well-conserved Gln in motif II/A5 (Gln^417^ of Acs1_Mt_). Asp at the last position plays a critical role in active-site architecture through a hydrogen bond network and in ATP binding and catalysis through key interactions with the hydroxyl groups of the ribose moiety. This Asp is invariant across the entire superfamily, consistent with a critical role in ATP binding and catalysis of the adenylate-forming first step of the reaction in all members.

## Figures and Tables

**Table 1:** Alignment of motif III residues in Acs sequences.

Consensus		**YXXGD**	
*M. thermoautotrophicus* Acs1	498	**Y**TA**GD**	503
*Salmonella enterica* Acs	496	**Y**FS**GD**	501
*Saccharomyces cerevisiae* Acs1	555	**Y**FT**GD**	560
*Methanosaeta concilii* Acs1	525	**Y**LA**GD**	530
*Methanosaeta concilii* Acs2	545	**Y**LA**GD**	550
*Methanosaeta concilii* Acs3	515	**Y**LA**GD**	520
*Methanosaeta concilii* Acs4	509	**Y**FS**GD**	514
*Methanosaeta concilii* Acs5	514	**Y**LA**GD**	519
*Homo sapiens* ACSM2A	442	WLL**GD**	446
*Alcaligenes *sp. AL3007 CBL	442	WLL**GD**	446
*Bacillus cereus* DltA	379	**Y**RT**GD**	383
		^ ∗ ∗^	

Positions within each sequence are shown, and conserved residues are indicated with an asterisk. *M. thermautotrophicus* Acs1, gi:82541817; *Salmonella enterica* Acs, gi:16767525; *Saccharomyces  cerevisiae* Acs1, gi:6319264; *Methanosaeta concilii* Acs1, gi:330506788; *Methanosaeta concilii* Acs2, gi:330506787; *Methanosaeta concilii* Acs3, gi:330506786; *Methanosaeta concilii* Acs4, gi:330506785; *Methanosaeta concilii* Acs5, gi:330508629; *Homo sapiens* ACSM2A, gi:58082049; *Alcaligenes *sp. AL3007 CBL, gi:197725159; *Bacillus cereus *DltA, gi:226887779.

**Table 2:** Kinetic parameters for ACS1_Mt_ wild-type and variant enzymes.

Enzyme	*k*_cat_	*K*_*m*_ acetate	*K*_*m*_ ATP	*K*_*m*_ CoA
	(sec^−1^)	(mM)	(mM)	(mM)
Acs1_Mt_	66.6 ± 0.9	3.5 ± 0.1	3.3 ± 0.2	0.19 ± 0.003
Tyr^498^Ala	1.6 ± 0.04	7.5 ± 0.6	1.7 ± 0.3	0.10 ± 0.004
Tyr^498^Phe		Inactive^a^		
Thr^499^Ala	0.8 ± 0.01	3.0 ± 0.01	1.7 ± 0.1	0.24 ± 0.01
Ala^500^Thr	1.5 ± 0.04	2.1 ± 0.2	3.6 ± 0.1	0.51 ± 0.04
Gly^501^Ala	0.3 ± 0.01	1.4 ± 0.1	1.7 ± 0.07	0.08 ± 0.002
Asp^502^Ala		Inactive^a^		
Asp^502^Glu		Inactive^a^		
Asp^502^Asn		Inactive^a^		

^a^Activity was tested over a wide range of concentrations for each substrate and at several enzyme concentrations, but no activity was observed.
